# Supplementary material for: Assessment of Human Health Risk Indices Due to Metal Contamination in the Surface Water of the Negro River Sub-Basin, Áncash
Source: Int J Environ Res Public Health. 2024 Jun 5;21(6):733. doi: 10.3390/ijerph21060733 (PMC11203810; doi:10.3390/ijerph21060733)
Supplement: Supplementary file 1 [file ijerph-21-00733-s001.zip › ijerph-2969488-supplementary.pdf]

Supplementary Material

Table S1. Distribution of sampling points in the Negro River sub-basin.

Sampling Point	UTM – DATUM WGS84 Zone 18 South			Site
	**East**	**North**	**Altitude**	
LTara1	245243	8942131	4509	Tarahua Lagoon
LTara2	244828	8941875	4493	Tarahua Lagoon
LTara3	244229	8941296	4493	Tarahua Lagoon
QArar	244414	8933087	4099	Araranca Stream
QOtut	244656	8934156	4071	Otuto Stream
QPuma	244170	8934256	4068	Pumahuaganga Stream
QPuyh	232379	8931485	3662	Puyhuan Stream
QQuil	243379	8932247	4132	Quilloc Stream
QRure1	243651	8941354	4474	Rurec Stream
QRure2	237859	8933083	3914	Rurec Stream
QRure3	237103	8932733	3911	Rurec Stream
QSNom2	238232	8933822	3964	Sin Nombre Stream 2
QSNom3	247597	8936268	4488	Sin Nombre Stream 3
QSNom4	247499	8935950	4448	Sin Nombre Stream 4
QSNom5	246729	8935602	4380	Sin Nombre Stream 5
QSNom7	245736	8935344	4264	Sin Nombre Stream 7
QUqui	240266	8931815	3989	Uquian Stream
RNegr	233102	8929794	3655	Negro River
ROlle	229715	8930436	3423	Olleros River

Figure S1. Instruction Protocol for Determining Inorganic Anions in Water Samples by Ion Chromatography (IC)

SECTOR ENERGÍA Y MINAS INGEMMET INSTITUTO GEOLÓGICO, MINERO Y METALÚRGICO	DETERMINATION OF INORGANIC ANIONS IN WATER SAMPLES BY ION CHROMATOGRAPHY	Code: M2-FP.4-037<br>Version: 01<br>Approval date: 2021-12-22<br>Page: 1

Instruction Protocol
(flowchart)

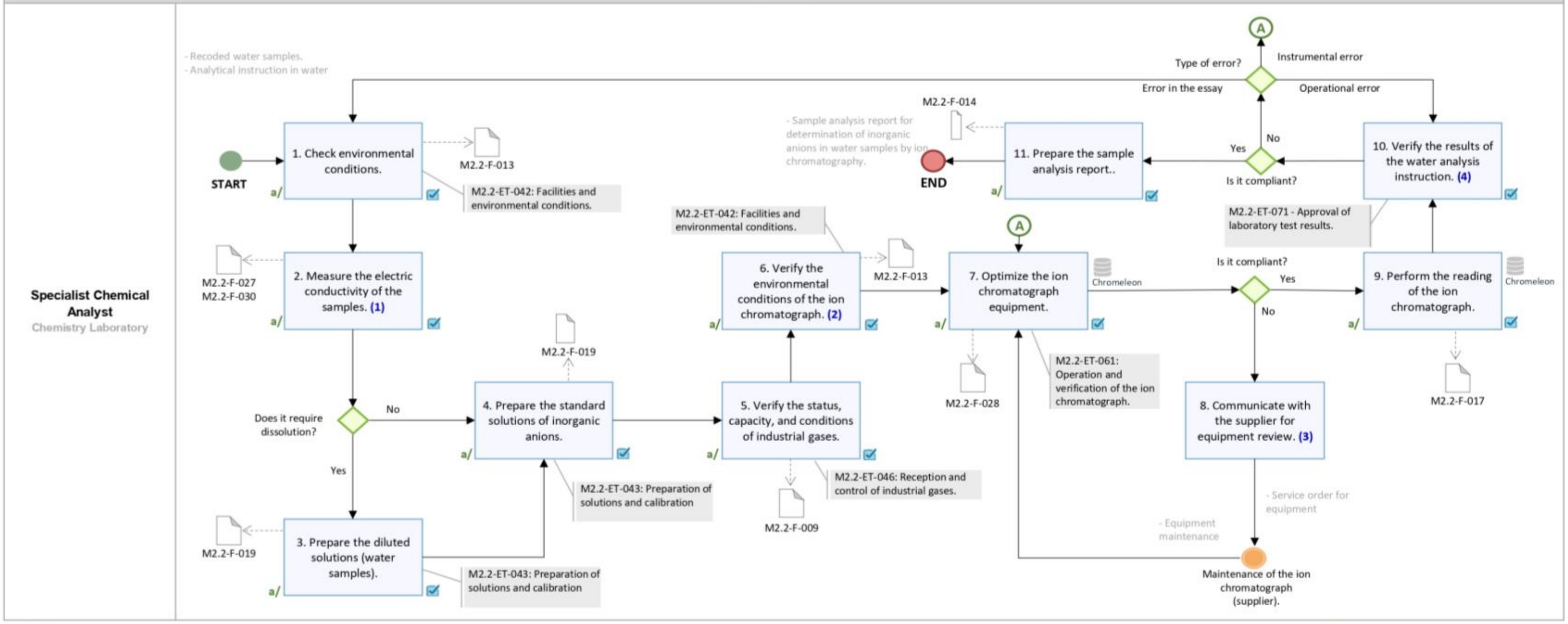

Notas

(1) Register according to the M2.2-F-027 format (Calibration control and verification of pH and conductivity), then proceed according to the technical specifications M2.2-F-030 (Measurement of pH, conductivity, and determination of total alkalinity in water samples).

(2) Referred to the area where the ion chromatograph equipment is located.

(3) Communication is made to the supplier via email to schedule corrective maintenance of the equipment. Then a requirement and a service order are generated through the hiring process.

(4) It must be verified that the deviations of the process blanks (<MDL), differences in duplicates (%RSD= max. 20%), and certified reference material (% recovery= 0 to 20%) are within the allowed limits.

S1.1.1.2. Document reception in office
S1.1.2.1. Document issuance in office

Registros de ejecución

a/ Format(s) associated with the task

Formats

- M2.2-F-009: Internal control of the entry and exit of industrial gases
- M2.2-F-013: Daily control of environmental condition
- M2.2-F-014: Sample analysis report
- M2.2-F-017: Control of sample reading.
- M2.2-F-019:Preparation of solutions.
- M2.2-F-027: Calibration control and verification of pH and conductivity.
- M2.2-F-028: Checklist for verification of the ion chromatograph equipment.
- M2.2-F-030: Determination of alkalinity in water samples.

Abbreviations

IC: Ion Chromatography
MDL: Method Detection Limit
%RSD: Relative Standard Deviation

Source: EPA Method 300.1 [7]

Figure S2. Instruction Protocol for Determining Trace Elements in Water Samples by Inductively Coupled Plasma Atomic Emission Spectrometry (ICP-AES)

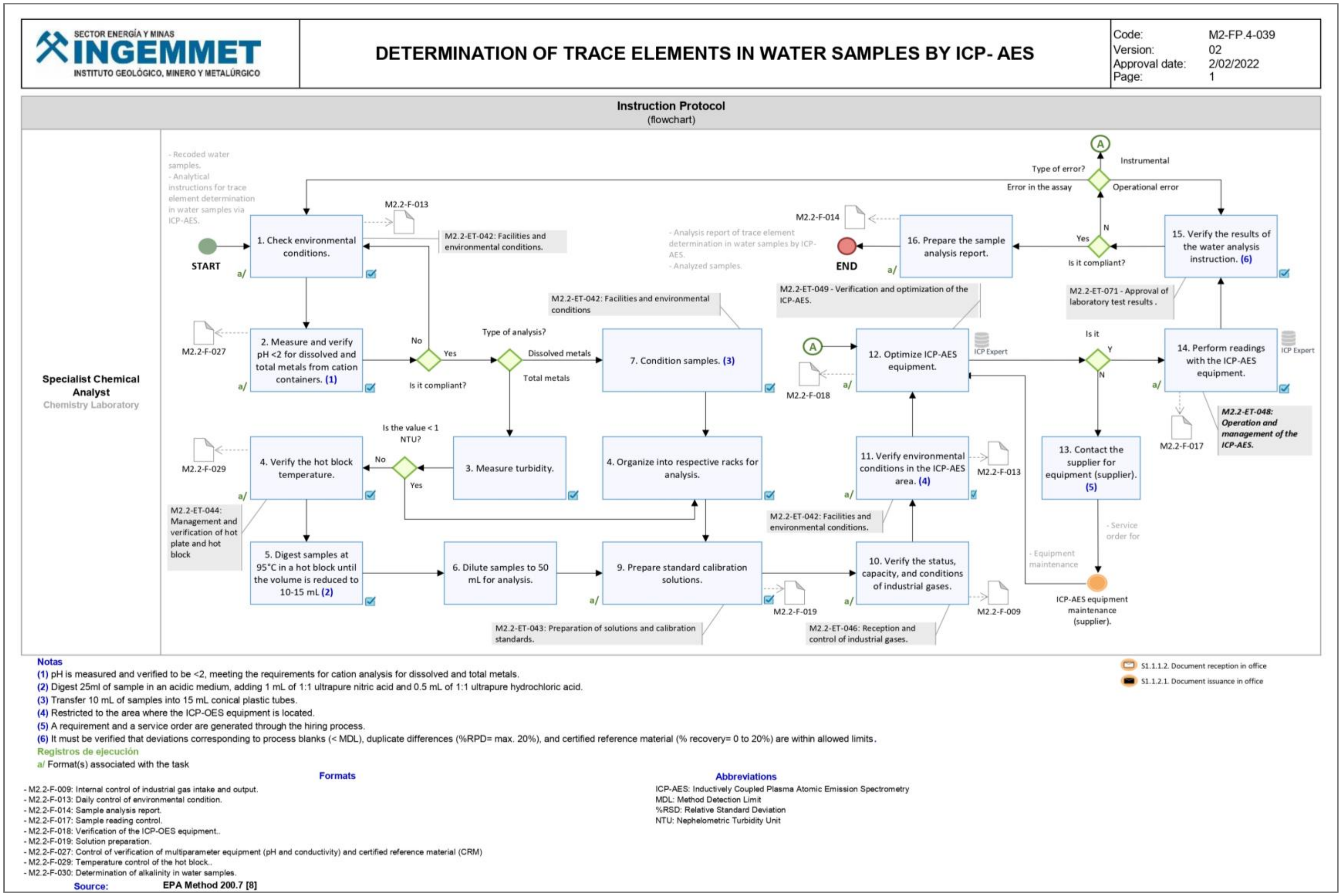

Figure S3. Instruction Protocol for Determining Trace Elements in Water Samples by Inductively Coupled Plasma Mass Spectrometry (ICP-MS)

SECTOR ENERGÍA Y MINAS INGEMMET INSTITUTO GEOLÓGICO, MINERO Y METALÚRGICO	DETERMINATION OF TRACE ELEMENTS IN WATER SAMPLES BY ICP-MS	Code: M2-FP.4-038<br>Version: 02<br>Approval date: 2/02/2022<br>Page: 1

Instruction Protocol
(flowchart)

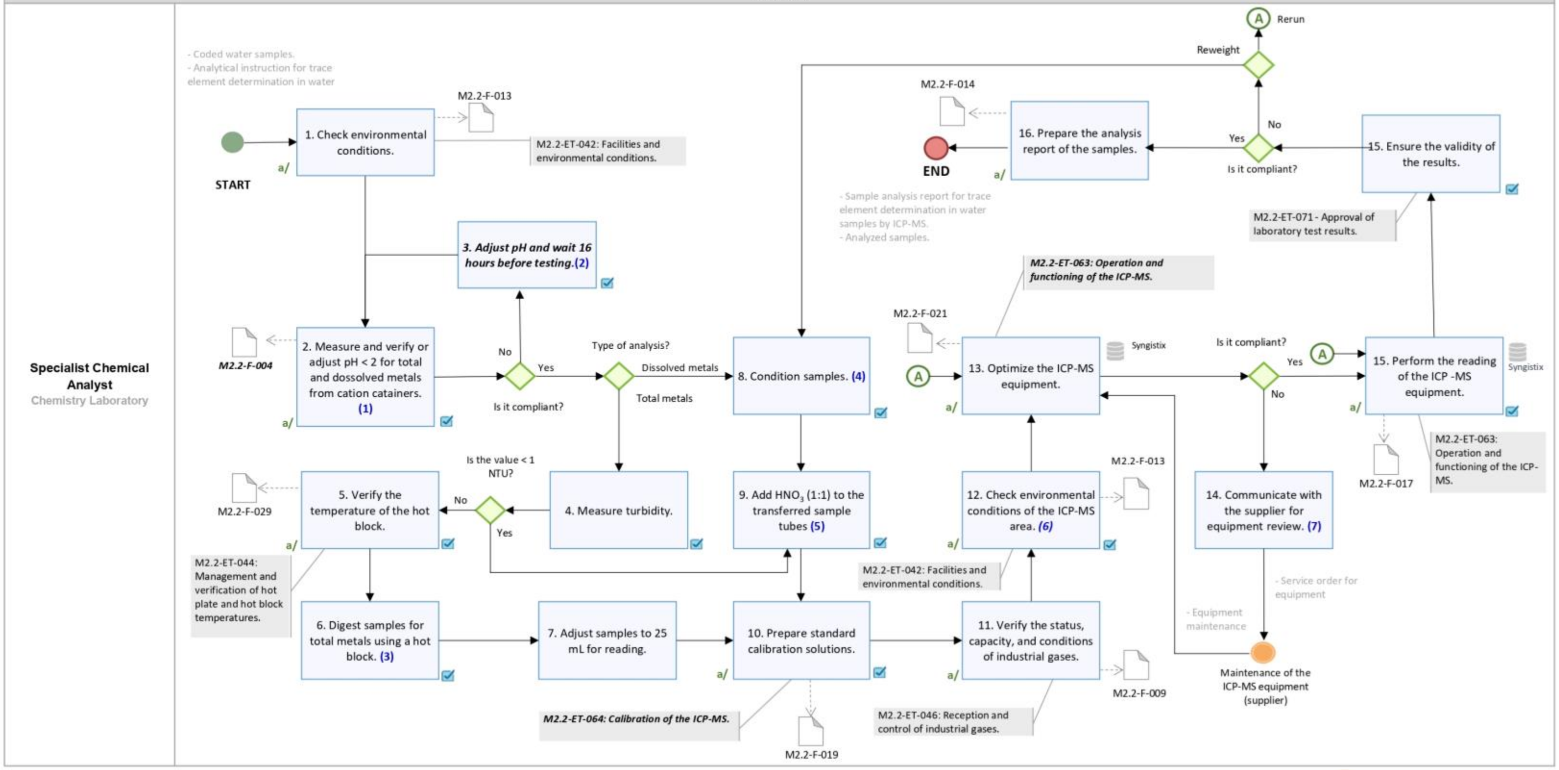

Notas
(1) It is measured and verified that the pH is <2.
(2) Adjust the pH when samples are submitted within two weeks after sampling.
(3) Transfer 25 ml of sample to a 50 ml plastic tube, adding 0.5 ml of ultrapure 1:1 nitric acid and 0.25 ml of ultrapure 1:1 hydrochloric acid; to make up to 5 mL volume.
(4) Transfer 10 ml of sample into 15 ml conical plastic tubes.
(5) Add 0.2 mL of ultrapure nitric acid (1:1), cap and shake.
(6) Referring to the area where the ICP-MS equipment is located.
(7) A requirement and a service order are generated through the hiring process.
Registros de ejecución
a/ Format(s) associated with the task

Formats
-M2.2-F-009: Internal control of entry and exit of industrial gases.
-M2.2-F-013: Daily control of environmental conditions.
-M2.2-F-014: Sample analysis report.
-M2.2-F-017: Control of sample reading.
-M2.2-F-019: Preparation of solutions.
-M2.2-F-021: Verification of ICP-MS equipment.
-M2.2-F-029: Temperature control of the hot block.

Abbreviations
-ICP-MS: Inductively Coupled Plasma Mass Spectrometer
-NTU: Nephelometric Turbidity Unit

S1.1.1.2. Document reception in office
S1.2.1. Document issuance in office

Source: EPA Method 200.8 [9]

Table S2. Standard Solutions for Calibration Curve.

Method	Element		Code	Lot N°	Brand	Expiration date	Concentration (μg/mL)
ICP-AES[a]	Iron	Fe	CGFE10	R2-FE693527	Inorganic Ventures	10/06/2024	10000
ICP-AES[a]	Sulfur	S	CGS10	N2-S673651	Inorganic Ventures	05/12/2022	10000
ICP-MS[b]	Aluminum	Al	CGAL10	R2-AL689264	Inorganic Ventures	22/02/2024	10000
ICP-MS[b]	Copper	Cu	CGCU1	P2-CU683968	Inorganic Ventures	02/11/2023	1000
ICP-MS[b]	Cobalt	Co	CGCO1	R2-CO690179	Inorganic Ventures	28/02/2024	1000
ICP-MS[b]	Lithium	Li	CGLI1	P2-LI675235	Inorganic Ventures	09/02/2023	1000
ICP-MS[b]	Manganese	Mn	CGMN1	R2-MN696895	Inorganic Ventures	24/09/2024	1000
ICP-MS[b]	Nickel	Ni	CGNI1	P2-NI680034	Inorganic Ventures	07/06/2023	1000
ICP-MS[b]	Lead	Pb	CGPB1	P2-PB676241	Inorganic Ventures	06/02/2023	1000
ICP-MS[b]	Zinc	Zn	CGZN1	R2-ZN693533	Inorganic Ventures	08/07/2024	1000
IC[c]	Chloride	Cl^-	ICCL1	P2-CL675597	Inorganic Ventures	30/01/2023	1000
IC[c]	Sulfate	SO_4^{2-}	ICS041	P2-SOX677315	Inorganic Ventures	03/04/2023	1000

[a] Determination of Metals and Trace Elements in water and wastes by Inductively Coupled Plasma-Atomic Emission Spectrometry (ICP-AES); EPA 200.7 REV.4.4, 1994

[b] Determination of trace Elements in water and wastes by Inductively Coupled Plasma-Mass Spectrometry (ICP-MS); EPA- Method 200.8 Rev. 5.4, 1994.

[b] Determination of Inorganic Anions by Ion Chromatography (IC); EPA 300.0, REV. 2.1, 1983.

Table S3. Standard Solutions for Quality Assurance of Results.

Method		Element	Code	Lot N°	Brand	Expiration date	Concentration (µg/mL)
ICP-AES[a]	ICP-OES tuning solution	50ug/ml: Al, As, Ba, Cd, Co, Cr, Cu, Mn, Mo, Ni, Pb, Se, Sr, Zn	IV-STOCK-24	T2-MEB720868	Inorganic Ventures	30/06/2027	50ug/ml: Al, As, Ba, Cd, Co, Cr, Cu, Mn, Mo, Ni, Pb, Se, Sr, Zn.
ICP-MS[b]	Multielement-2	Al, As, Ba, Be, Bi, Ca, Cs, Ga, In, K, Li, Mg, Na, Rb, Se, Sr	CCS-4	T2-MEB719528	Inorganic Ventures	01/06/2027	100
ICP-MS[b]	Multielement-4	Ag, Cd, Co, Cr, Cu, Fe, Hg, Mn, Ni, Pb, Tl, V, Zn.	CCS-6	P2-MEB681853	Inorganic Ventures	02/07/2023	100
ICP-MS[b]	Multielement-7	As, B, Ba, Be, Bi, Cd, Ga, In, Pb, Sb, Se, Tl, V	CMS-4	P2-MEB680583	Inorganic Ventures	23/05/2023	10
ICP-MS[b]	Multielement-8	Ag, Al, Ca, Co, Cr, Cs, Cu, Fe, K, Li, Mg, Mn, Na, Ni, Rb, Sr, Zn	CMS-5	R2-MEB693959	Inorganic Ventures	18/06/2024	10
ICP-MS[b]	Multielement-9	Ag, Al, B, Ba, Bi, Ca, Cd, Co, Cr, Cu, Fe, Ga, In, K, Li, Mg, Mn, Na, Ni, Pb, Sr, Tl, Zn	IV-STOCK-4	T2-MEB713995	Inorganic Ventures	12/01/2027	1000
ICP-MS[b]	Multielement-10	Al, B, Ba, Be, Bi, Ca, Cd, Co, Cr, Cu, Fe, Ga, K, Li, Mg, Mn, Na, Ni, Pb, Se, Sr, Te, Tl, Zn	IV-STOCK-8	P2-MEB680683	Inorganic Ventures	23/05/2023	100
ICP-MS[b]	Multielement-12	Ca, Fe, K, Mg, Na	IV-STOCK-35	S2-MEB710811	Inorganic Ventures	07/10/2023	1000
IC[c]	Multielement-1	QCP-QCS-5	QCP-QCS-5	P2-MEB676890	Inorganic Ventures	16/02/2023	1000
IC[c]	Multielement-2	1C-FAS-1A	1C-FAS-1A	P2-MEB680049	Inorganic Ventures	17/03/2023	-
IC[c]	Subrogado	DCA	ICDCA-S	R2-OI693532	Inorganic Ventures	01/07/2024	500

[a] Determination of Metals and Trace Elements in water and wastes by Inductively Coupled Plasma-Atomic Emission Spectrometry (ICP-AES); EPA 200.7 REV.4.4, 1994

[b] Determination of trace Elements in water and wastes by Inductively Coupled Plasma-Mass Spectrometry (ICP-MS); EPA- Method 200.8 Rev. 5.4, 1994.

[b] Determination of Inorganic Anions by Ion Chromatography (IC); EPA 300.0, REV. 2.1, 1983.

Table S4. Categorization of the Heavy Metal Pollution Index (HPI).

HPI	Grado de Contaminación [1-3]
HPI < 90	Low Pollution
90 ≤ HPI < 150	Medium Pollution
HPI ≥ 150	High Pollution

Table S5. Sampling Points Evaluated with ECA-Peru.

Sampling Point	ECA-Peru [4]
LTara1	ECA.4.E1
LTara2	ECA.4.E1
LTara3	ECA.4.E1
QArar	ECA.4.E2
QOtut	ECA.4.E2
QPuma	ECA.4.E2
QPuyh	ECA.1.A2
QQuil	ECA.4.E2
QRure1	ECA.4.E2
QRure2	ECA.1.A2
QRure3	ECA.1.A2
QSNom2	ECA.1.A2
QSNom3	ECA.4.E2
QSNom4	ECA.4.E2
QSNom5	ECA.4.E2
QSNom7	ECA.4.E2
QUqui	ECA.1.A2
RNegr	ECA.1.A2
ROlle	ECA.1.A2

ECA.1.A2.: Population and recreational category for waters that can be treated with conventional treatment.

ECA.4.E1.: Aquatic environment conservation category for lagoons.

ECA.4.E2.: Aquatic environment conservation category for rivers.

Table S6. Results of In Situ Parameters.

Sampling Points	pH	DO	EC	TDS	Salinity	Temperature
	pH unit	mg/L	μS/cm	Ppm	PSU	°C
LTara1	3.212	7.218	1410.8	705.6	0.700	1.268
LTara2	3.642	7.492	480.2	240.4	0.230	4.364
LTara3	2.772	6.490	1093.8	553.0	0.548	8.708
QArar	6.554	7.010	82.8	41.4	0.040	7.262
QOtut	2.708	7.418	1175.2	587.0	0.590	7.054
QPuma	2.990	6.438	1848.6	924.6	0.948	13.092
QPuyh	6.912	6.446	105.4	49.0	0.050	14.350
QQuil	6.192	7.784	69.4	34.4	0.030	4.066
QRure1	6.210	6.538	84.0	42.0	0.040	8.310
QRure2	2.998	4.972	859.4	430.0	0.430	11.388
QRure3	3.072	5.400	851.8	426.0	0.420	11.260
QSNom2	6.488	6.814	111.8	56.0	0.050	9.682
QSNom3	2.756	5.742	1557.2	778.80	0.790	13.250
QSNom4	6.646	5.570	73.8	36.8	0.030	13.558
QSNom5	2.890	5.534	1271.6	636.0	0.640	10.352
QSNom7	2.790	6.334	1346.8	672.4	0.680	11.816
QUqui	2.772	7.548	1279.4	639.8	0.640	4.802
RNegr	2.896	7.482	1006.8	503.0	0.500	9.170
ROlle	2.960	7.368	997.6	499.0	0.500	12.192
ECA1.A2 [4]	5.5 - 9	≥5	1600	1000	**	Δ 3
ECA4.E1 [4]	6.5 – 9	≥5	1000	**	**	Δ 3
ECA4.E2 [4]	6.5 – 9	≥5	1000	**	**	Δ 3
WHO [5]	6.5-8.5	**	**	**	**	**
US EPA [6]	6.5-8.5	**	**	500	**	**

* Not applicable for the subcategory.

** No corresponding value exists for the subcategory

Table S7. Results of Metals, Ions, and Turbidity in Laboratory

Parameters	Cl^-	SO_4^{2-}	Fe	Al	Cu	Co	Li	Mn	Ni	Pb	S	Zn	Turbidity
Units	mg/L	mg/L	mg/L	mg/L	mg/L	mg/L	mg/L	mg/L	mg/L	mg/L	mg/L	mg/L	NTU
MDL	<0.2	<1	<0.01	<0.005	<0.0005	<0.0002	<0.0004	<0.0002	<0.0007	<0.0005	<0.3	<0.001	<0.05
LTara1	1.8	297.7	51.66	3.843	0.007	0.060	0.036	1.434	0.109	0.003	99.8	0.351	0.071
LTara2	<0.2	97.8	6.85	1.916	0.006	0.010	0.011	0.555	0.019	0.004	40.4	0.131	0.084
LTara3	0.4	170.4	17.54	3.761	0.008	0.057	0.027	1.288	0.099	0.002	69.4	0.281	0.078
QArar	<0.2	12.8	0.28	0.029	0.001	<0.0002	0.007	0.019	0.002	0.001	4.4	0.016	0.076
QOtut	1.5	208.8	36.64	7.862	0.008	0.124	0.037	2.307	0.162	0.002	94.1	0.414	0.078
QPuma	0.6	493.8	160.85	7.973	0.006	0.062	0.037	1.614	0.198	0.001	191.4	0.554	0.101
QPuyh	0.8	2.8	0.20	0.049	0.002	<0.0002	<0.0004	0.004	<0.0007	0.002	1.4	0.034	0.095
QQuil	<0.2	12.1	0.17	0.042	0.003	0.001	0.007	0.030	0.002	<0.0005	4.7	0.013	0.106
QRure1	<0.2	10.4	0.11	0.047	0.002	<0.0002	<0.0004	0.003	<0.0007	0.001	4.1	0.016	0.086
QRure2	0.6	131.9	5.62	1.968	0.004	0.027	0.015	0.608	0.046x	0.001	35.2	0.141	0.096
QRure3	0.6	146.2	8.37	3.128	0.006	0.043	0.025	0.990	0.075	0.002	52.5	0.237	0.125
QSNom2	<0.2	1.6	0.13	0.079	0.001	<0.0002	0.001	0.005	<0.0007	0.001	0.9	0.046	0.323
QSNom3	1.0	301.8	65.37	10.573	0.008	0.181	0.301	3.258	0.237	0.002	131.4	0.555	0.298
QSNom4	<0.2	4.8	0.96	0.022	0.001	0.001	0.003	0.050	<0.0007	0.001	2.4	0.008	0.192
QSNom5	0.6	244.0	36.26	7.299	0.004	0.121	0.068	2.042	0.156	0.001	85.6	0.332	0.206
QSNom7	0.7	249.9	44.30	9.017	0.006	0.146	0.027	2.531	0.190	0.001	103.4	0.422	0.165
QUqui	1.1	253.7	61.04	6.421	0.005	0.073	0.032	1.601	0.140	0.001	104.8	0.373	0.087
RNegr	0.5	170.0	24.53	4.535	0.006	0.052	0.027	1.168	0.097	0.002	74.4	0.270	0.093
ROlle	1.3	167.3	22.47	4.251	0.009	0.048	0.034	1.100	0.088	0.004	63.5	0.249	0.075
Field Blank	<0.2	<1	<0.01	<0.005	<0.0005	<0.0002	<0.0004	<0.0002	<0.0007	<0.0005	<0.3	<0.001	<0.05
Transport Blank	<0.2	<1	<0.01	<0.005	<0.0005	<0.0002	<0.0004	<0.0002	<0.0007	<0.0005	<0.3	<0.001	<0.05
QRure2 - dup	0.7	135	5.3	1.938	0.004	0.027	0.015	0.596	0.045	0.001	30.5	0.139	0.101
RNegr -dup	0.5	171	23.03	4.571	0.006	0.052	0.027	0.168	0.097	0.001	70.7	0.270	0.099
ECA1.A2 [4]	250	500	1	5	2	**	**	0.4	*	0.05	**	5	100
ECA4.E1 [4]	**	**	**	**	0.1	**	**	**	0.052	0.0025	**	0.12	*
ECA4.E2 [4]	**	**	**	**	0.1	**	**	**	0.052	0.0025	**	0.12	*
WHO [5]	250	250	0.3	0.2	1.0	**	**	0.02	0.07	0.01	**	3	5
US EPA [6]	250	250	0.3	0.2	1.0	**	**	0.05	0.1	0	**	5	5

* Not applicable for the subcategory.

** No corresponding value exists for the subcategory

Table S8. Result and categorization of the HPI for each sampling point.

Sampling Point	Heavy Metal Pollution Index (HPI) [5-8]	Category
LTara1	578.912	High pollution
LTara2	139.331	Medium pollution
LTara3	268.705	High pollution
QArar	12.161	Low pollution
QOtut	536.677	High pollution
QPuma	1,627.805	High pollution
QPuyh	15.533	Low pollution
QQuil	4.847	Low pollution
QRure1	12.311	Low pollution
QRure2	107.218	Medium pollution
QRure3	165.098	High pollution
QSNom2	12.337	Low pollution
QSNom3	853.902	High pollution
QSNom4	14.786	Low pollution
QSNom5	507.821	High pollution
QSNom7	623.323	High pollution
QUqui	705.203	High pollution
RNegr	340.251	High pollution
ROlle	332.021	High pollution

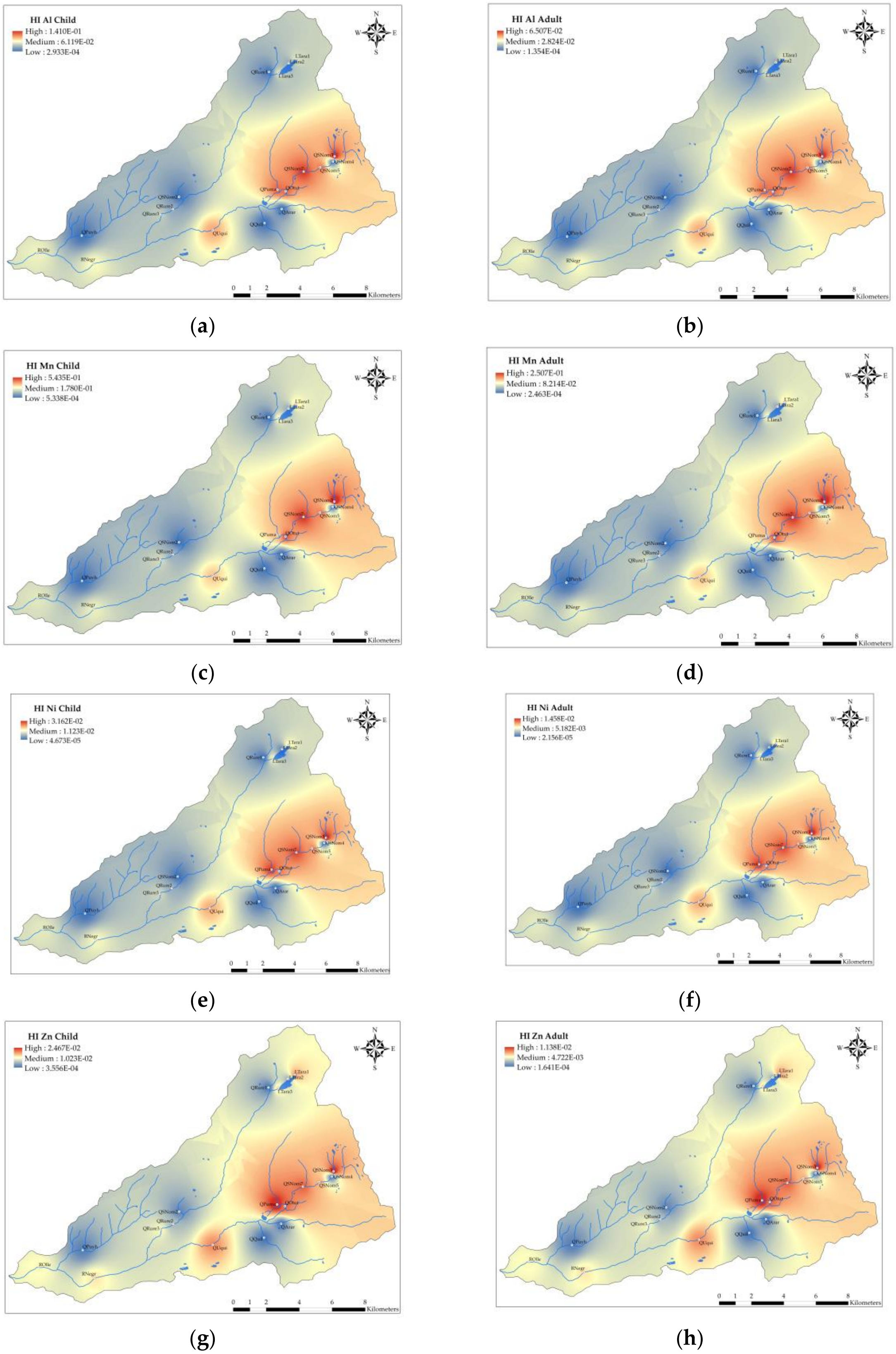

Figure S4. Spatial Distribution of HI for Al, Mn, Ni, and Zn (a) HI for Al in children; (b) HI for Al in adults; (c) HI for Mn in children; (d) HI for Mn in adults; (e) HI for Ni in children; (f) HI for Ni in adults; (g) HI for Zn in children; (h) HI for Zn in adults.

References

1. Kwaya, M.; Hassan, H.; Abdullahi, I.; Yelwa, N.A.; Ibrahim, H.; Grema, H.; Mohammed, D.; Halilu, F.; M.A, K. Heavy Metals Pollution Indices and Multivariate Statistical Evaluation of Groundwater Quality of Maru town and environs. *J. Mat. Environ. Sci.* **2019**, 10, 32-44.
2. Karunanidhi, D.; Aravinthasamy, P.; Subramani, T.; Setia, R. Effects of COVID-19 pandemic lockdown on microbial and metals contaminations in a part of Thirumanimuthar River, South India: A comparative health hazard perspective. *Journal of Hazardous Materials* **2021**, 416, 125909, doi:10.1016/j.jhazmat.2021.125909.
3. Chakraborty, B.; Bera, B.; Adhikary, P.P.; Bhattacharjee, S.; Roy, S.; Saha, S.; Sengupta, D.; Shit, P. Effects of COVID-19 Lockdown and Unlock on the Health of Tropical Large River with Associated Human Health Risk. *Environmental Science and Pollution Research* **2022**, 29, 37041–56, doi:10.1007/s11356-021-17881-w.
4. Ministerio del Ambiente. *Estándares de Calidad Ambiental (ECA), Decreto Supremo N 004-2017-MINAM-Perú (10 p.)*; Ministerio del Ambiente: Lima, Perú, 2017.
5. World Health Organization. *Guidelines for drinking-water quality: fourth edition incorporating the first and second addenda. Ginebra: World Health Organization*; World Health Organization: Geneva, Switzerland, 2022.
6. US EPA. National Primary Drinking Water Regulations. Available online: https://www.epa.gov/ground-water-and-drinking-water/national-primary-drinking-water-regulations (accessed on 23 February 2023)
7. US EPA. EPA Method 300.1, Revision 1.0: Determination of Inorganic Anions in Drinking Water by Ion Chromatography. Available online: https://www.epa.gov/esam/epa-method-3001-revision-10-determination-inorganic-anions-drinking-water-ion-chromatography (accessed on 22 February 2023).
8. US EPA. EPA Method 200.7: Determination of Metals and Trace Elements in Water and Wastes by Inductively Coupled Plasma-Atomic Emission Spectrometry. Available online: https://www.epa.gov/esam/method-2007-determination-metals-and-trace-elements-water-and-wastes-inductively-coupled (accessed on 22 February 2023).
9. US EPA. EPA Method 200.8: Determination of Trace Elements in Waters and Wastes by Inductively Coupled Plasma-Mass Spectrometry. Available online: https://www.epa.gov/esam/epa-method-2008-determination-trace-elements-waters-and-wastes-inductively-coupled-plasma-mass (accessed on 23 February 2023)
10. Mohan, S.V.; Nithila, P.; Reddy, S.J. Estimation of heavy metals in drinking water and development of heavy metal pollution index. *J. Environ. Sci. Health Part A* **1996**, *31*, 283–289, doi:10.1080/10934529609376357
11. Kwaya, M.; Hassan, H.; Abdullahi, I.; Yelwa, N.A.; Ibrahim, H.; Grema, H.; Mohammed, D.; Halilu, F.; M.A, K. Heavy Metals Pollution Indices and Multivariate Statistical Evaluation of Groundwater Quality of Maru town and environs. *J. Mat. Environ. Sci.* **2019**, 10, 32-44.
12. Karunanidhi, D.; Aravinthasamy, P.; Subramani, T.; Setia, R. Effects of COVID-19 pandemic lockdown on microbial and metals contaminations in a part of Thirumanimuthar River, South India: A comparative health hazard perspective. *Journal of Hazardous Materials* **2021**, 416, 125909, doi:10.1016/j.jhazmat.2021.125909.
13. Chakraborty, B.; Bera, B.; Adhikary, P.P.; Bhattacharjee, S.; Roy, S.; Saha, S.; Sengupta, D.; Shit, P. Effects of COVID-19 Lockdown and Unlock on the Health of Tropical Large River with Associated Human Health Risk. *Environmental Science and Pollution Research* **2022**, 29, 37041–56, doi:10.1007/s11356-021-17881-w.
